# Two de novo GJA1 mutation in two sporadic patients with erythrokeratodermia variabilis et progressiva

**DOI:** 10.1002/mgg3.670

**Published:** 2019-03-29

**Authors:** Changxing Li, Jingyao Liang, Pingjiao Chen, Kang Zeng, Rujun Xue, Xin Tian, Liuping Liang, Qi Wang, Minglan Shi, Xibao Zhang

**Affiliations:** ^1^ Department of Dermatology Nanfang Hospital, Southern Medical University Guangzhou China; ^2^ Department of Dermatology Guangzhou Institute of Dermatology Guangzhou China

**Keywords:** Connexins 43, Erythrokeratodermia variabilis et progressiva, Gap junction alpha 1 gene

## Abstract

**Background:**

Erythrokeratodermia variabilis et progressiva (EKVP, OMIM 133200) is a rare hereditary disorder characterized by varies from transient, fast moving erythema to persistent brown hyperkeratotic plaques. Recently, mutations in the genes gap junction alpha 1 gene (GJA1), GJB3, and GJB4 have been reported to cause EKVP. Here, we report the identification of two de novo missense mutations in the GJA1 gene in two unrelated individuals with EKVP.

**Methods:**

The patients and his family members were subjected to mutation detection in the candidate gene GJA1, GJB3, and GJB4 by Sanger sequencing. The expression of connexin (Cx) 43 was detected by immunohistochemistry and immunofluorescence (IF) studies in the lesions.

**Results:**

A 12‐year‐old boy presented with multiple hyperkeratotic plaques on the face, neck, elbows, wrists, limbs, knees, inguinal region, hands, and feet. A 7‐year‐old girl presented with symmetrical erythematous, plaques on the hands, feet, wrists, and ankles. A novel heterozygous missense mutation c.848C > T (p.P283L) in exon 2 of the GJA1 gene was identified in both patients. A novel heterozygous missense mutation c.869C > A (p.T290N) in exon 2 of the GJA1 gene was also identified in the boy. These mutations were not found in the unaffected family members and 100 normal controls. In the patients’ lesions, Cx43 protein was located to the cytomembrane and cytoplasm in the stratum corneum, and granular layer. Compound heterozygous mutations in the boy showed a more severe clinical phenotype and cytoplasmic mislocalization.

**Conclusions:**

The novel mutations c.848C > T (p.P283L) and c.869C > A(p.T290N) arose de novo and were considered as the cause of two Chinese EKVP. GJA1 P283L and T290N mutations lead to Cx43 protein cytoplasmic mislocalization. Our finding expands the mutant spectrum of GJA1 gene and adds new understanding of the genotype‐phenotype correlation.

## 1 INTRODUCTION

Erythrokeratodermia variabilis et progressiva (EKVP, Online Mendelian Inheritance in Man [OMIM] no. 133200) is rare congenital skin disorder that are characterized by limited erythematous, hyperkeratotic plaques to severe progressive symmetrical erythrokeratodermia (Boyden et al., 2015). Clinical observations revealed no differences between males and females, and no association with malignancy. An autosomal dominant inheritance is usually seen in EKVP, although there are reports of sporadic patients as well as autosomal recessive inheritance (Boyden et al., 2015; Wang et al., 2011). The genetic basis of EKVP has also been explored. Causal mutations have been detected in the GJA1, GJB3, and GJB4 genes encoding connexins (Cx) 43, 31, and 30.3, respectively (Boyden et al., 2015; Fuchs‐Telem et al., 2011; Kokotas, Papagiannaki, Grigoriadou, & Katsarou, 2012; Macari et al., 2000; Morley et al., 2005; Richard et al., 1998; Scott, O'Toole, Mohungoo, & Kelsell, 2011; Wang et al., 2011). In this study, we report the identification of two de novo missense mutations in the GJA1 gene in two unrelated individuals with EKVP. Cx43 protein did not localize to the intracellular junctions, demonstrating cytoplasmic localization. The cytoplasmic mislocalization showed a more severe phenotype in the boy than that in the girl. This report broadens the spectrum of GJA1 gene mutations implicated in EKVP pathogenesis and adds new understanding of the genotype‐phenotype correlation.

## 2 MATERIALS AND METHODS

### 2.1 Subjects

Two affected individuals and their families were recruited from Department of Dermatology, Nanfang Hospital, Southern Medical University, China. Human samples used in this study were obtained in accordance with the principles expressed in the Declaration of Helsinki, and was approved by the Institutional Review Board from Nanfang Hospital, Southern Medical University. Blood and skin biopsy sample collection procedures used were in accordance with institutional and national ethical standards of human experimentation and all participants provided written informed consent.

### 2.2 Patient 1

**Figure 1:**
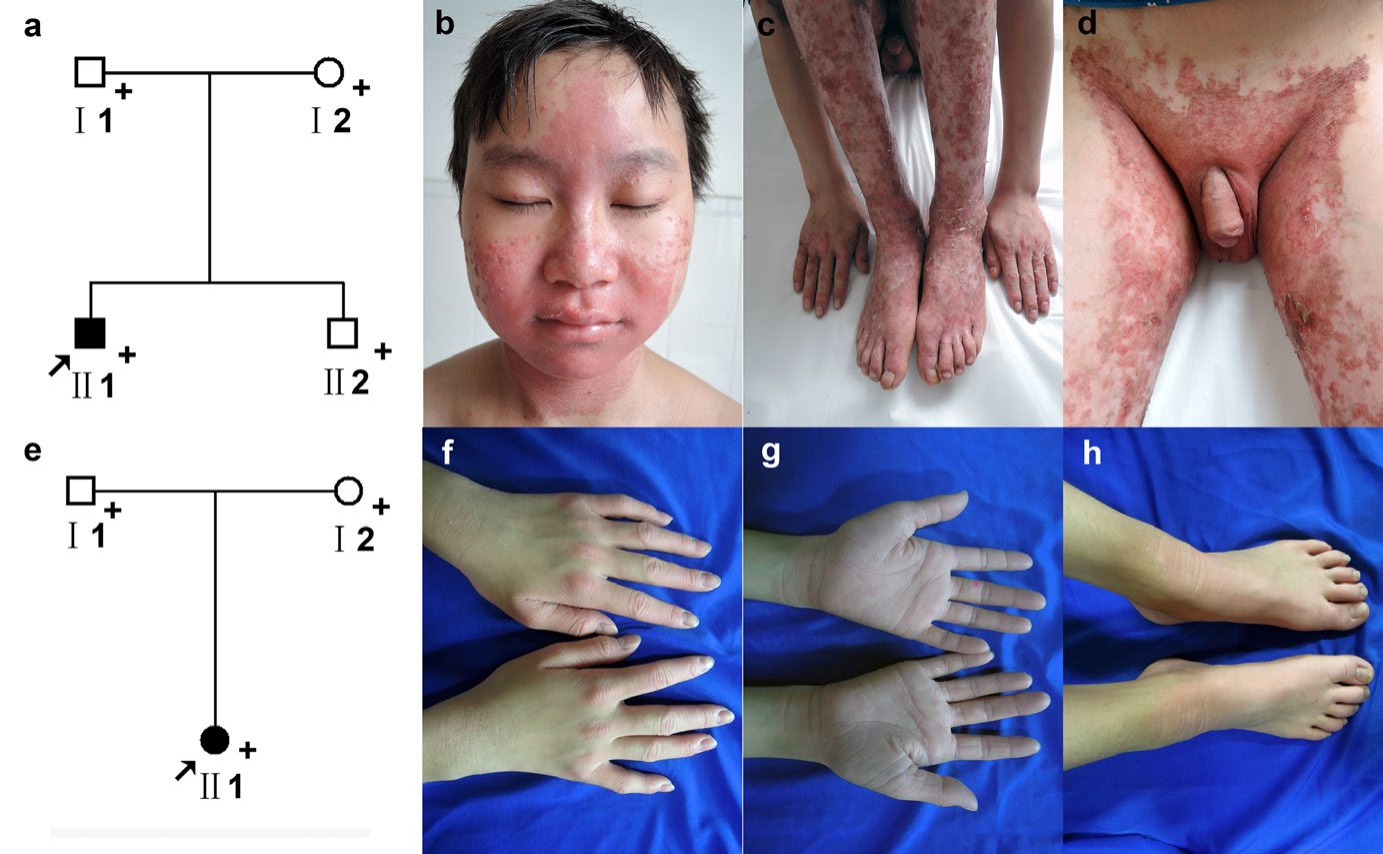
Family pedigree and clinical features of EKVP. (a) Pedigree was constructed for the four‐member family with EKVP. Squares and circles indicate males and females, respectively. Filled symbols indicate affected individuals. Arrow indicates the proband. “+” in pedigree indicates those who are subjected to Sanger sequencing. (b) Fixed, finely scaly, symmetrical erythematous keratotic erythema, plaques on the face, neck, elbows, wrists, limbs, (c) knees, (d) inguinal region of patient 1. (e) Pedigree was constructed for the three‐member family with EKVP. Squares and circles indicate males and females, respectively. Filled symbols indicate affected individuals. Arrow indicates the proband. “+” in pedigree indicates those who are subjected to Sanger sequencing. (f, g) Fixed, finely, symmetrical erythematous keratotic plaques on the hands, (h) feet of patient 2

A 12‐year‐old boy (Ⅱ1) (Figure 1a) presented with multiple lesions on the face, neck, elbows, wrists, limbs, knees, inguinal region, hands, and feet for 12 years. At one month of age, he developed symmetrical erythematous on the hands and feet, and progressive thickening of the palms and soles. By age 8 years, hyperkeratotic plaques appeared, affecting the face, neck, elbows, wrists, limbs, knees, inguinal region, hands, and feet. Lesions tend to became worse in summer and to improve in winter. Hair and teeth were not affected. Cutaneous examination showed fixed, finely scaly, symmetrical erythematous keratotic erythema, plaques on the face (Figure 1b), neck, elbows, wrists, limbs, knees (Figure 1c), inguinal region (Figure 1d), hands, and feet. The plaques were thicker on the elbows and there was a well‐defined, brownish‐colored hyperpigmentation halo on the inguinal region. His parents were not consanguineous. His parents and brother did not show any erythrokeratodermia variabilis related abnormality.

### 2.3 Patient 2

A 7‐year‐old girl (Ⅱ1) (Figure 1e) presented with multiple lesions on the hands, feet, wrists, and ankles for 6 years. She had erythematous on the hands and feet at the age of 10 months. She then developed symmetrical erythematous on the hands and feet, as well as progressive keratotic erythema, plaques on the dorsal hands, dorsal feet, wrists, and ankles. Lesions tend to become worse in summer and to improve in winter. Hair and teeth are not affected. Her parents were not consanguineous. Cutaneous examination showed fixed, finely, symmetrical erythematous, plaques on the hands (Figure 1f,g), feet (Figure 1h), wrists, and ankles. Her parents did not show any similar abnormality.

### 2.4 Polymerase chain reaction (PCR) and sequencing

Genomic DNA was isolated from peripheral blood of the patients using a Qiagen kit (Qiagen, Hilden, Germany). In addition, genomic DNA of unaffected family members and 100 unrelated healthy individuals was extracted as a control. All coding exons of the GJA1, GJB3, and GJB4 genes together with boundary exon–intron sequences were amplified using as previous study (Boyden et al., 2015; Kokotas et al., 2012; Scott et al., 2011). PCR of gene was carried out in a 30 µl total volume, containing 20 ng genomic DNA, 10 mM Tris–HCl (pH 8.3), 50 mM KCl, 3.0 mM MgCl_2_, 0.01% gelatine, 2 µl dNTPs, 10 pmol of each primer 1 µl, 0.2 U DNA Taq Polymerase (Takara, Japan), add ddH_2_O to 20.8 µl. The PCR program was set as below: HotStarsTaq activation at 96℃ for 5 min, followed by 30 cycles, each having denaturation at 96℃ for 20 s, annealing at 55℃ for 60 s and extension at 72℃ for 60 s, and the final extension was 72℃ for 5 min. PCR products were directly sequenced on an ABI 3730XL DNA Analyzer (Applied Biosystems, Foster City, CA). Direct sequencing of the PCR products was performed with a BigDye Direct Cycle Sequencing Kit (Applied Biosystems) and analyzed on the ABI 3,130 genetic analyzer (Applied Biosystems). Sequence comparisons and analysis were performed by Phred‐Phrap‐Consed program, version 12.0.

### 2.5 Immunohistochemistry

Four‐micrometer paraffin‐embedded sections of EKVP tissue were submitted to immunohistochemical processing by the streptavidin–biotin technique. The sections were performed by immersion in a modified pH 6.0 citrate buffer for 20 min in a steamer and subjected to incubation in 3% H_2_O_2_ for 10 min to block endogenous peroxidase activity. The sections were washed with phosphate‐buffered saline (PBS) three times and incubated with 20% normal goat serum at the room temperature for 30 min and further with a monoclonal anti‐Cx 43 antibody (1:50, ab66151, Abcam, Cambridge, England) at a dilution of 1:100 at 4°C overnight. The sections were washed with PBS three times and then incubated with a secondary antibody (DAKO company, Carpentaria, CA) at 37°C for 30 min and subsequently with a ChemMate™ EnVision™ Detection Kit (DAKO company). After that, the sections were stained with 3,3’‐diaminobenzidine (DAB) solution and counterstained with hematoxylin briefly and then mounted with a coverslip.

### 2.6 Immunofluorescence

Immunofluorescence studies were performed on 4‐µm paraffin‐embedded sections of EKVP tissue submitted from the biopsy. Slides were first deparaffinized and rehydrated, then placed in PBS for 10 min at room temperature. Sections were digested with 0.05% proteinase (Sigma, St. Louis, MO) in PBS at room temperature for 30 min. After two rinses in PBS, the slides were then fixed in 95% ethanol for 10 min and then rinsed twice in PBS for 5 min each before staining. The primary antibody Cx43 (1:25, ab66151, Abcam, Cambridge, UK) was applied to the slides and then diluted in blocking solution overnight at 4°C. Incubation with secondary antibodies (donkey anti‐rabbit‐488, 1:200, ab150065, Abcam, Cambridge, UK) diluted in 1% BSA in PBS was then performed for 1 hr at RT in the dark. This was followed by incubation with 0.5 μg/ml 4',6‐diamino‐2‐phenylindole (DAPI) (Sigma) and coverslipping with glycergel mounting medium.

## 3 RESULTS

### 3.1 Histopathological examination and laboratory examination

#### 3.1.1 Patient 1

**Figure 2:**
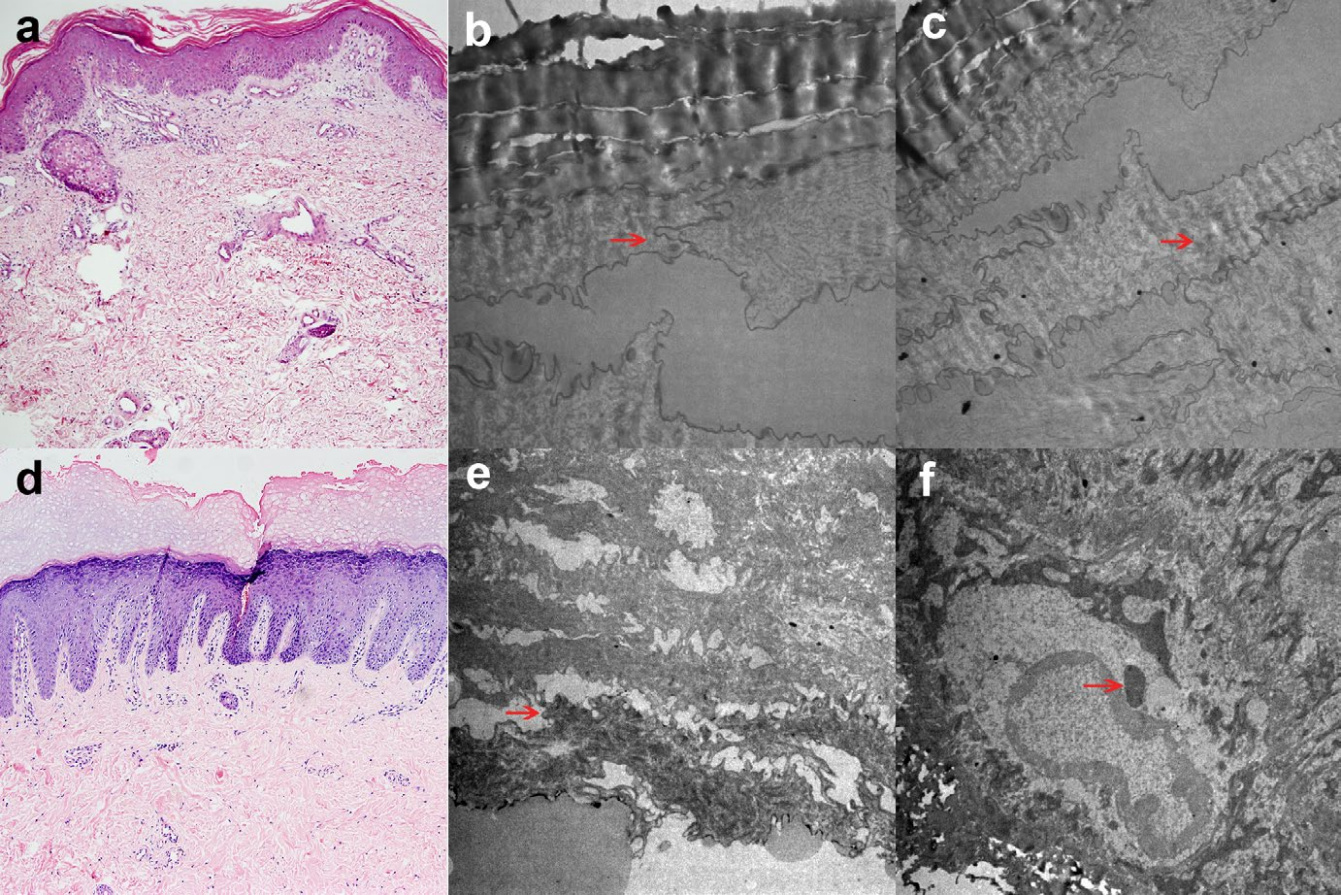
Histologic features of EKVP. (a) Histology of affected skin in patient 1 revealed papillomatosis, acanthosis, hypergranulosis, compact orthohyperkeratosis, parakeratosis, and follicular plugging (×100, H&E stain). (b) The electron microscope (EM) showed stratum corneum thickening, filaments tearing (red arrowheads) (×10,000), (c) and filaments of interstellar clouds (red arrowheads) (×8,000). (d) Histology of affected skin in patient 2 revealed compact orthohyperkeratosis, vacuolated granular cells with sparse keratohyalin granules, acanthosis, papillomatosis and perivascular infiltrates (×100, H&E stain). (e) The EM showed stratum corneum thickening, filaments tearing (red arrowhead) (×8,000), (f) the cytoplasm and nucleus edema of hyaline layer cells, superficial granular cells containing characteristic intranuclear granules (red arrowhead) (×10,000)

Histology of affected skin revealed only nonspecific findings: papillomatosis, acanthosis, hypergranulosis, compact orthohyperkeratosis, parakeratosis, and follicular plugging (Figure 2a). Complete blood count, urinalysis, fasting blood sugar, liver and renal function, complement, and immunoglobulin levels were within normal limits and serologic tests for syphilis and HIV were negative. The electron microscope (EM) showed stratum corneum thickening, filaments tearing, and filaments of interstellar clouds (Figure 2b,c) (red arrowheads). The cornified cell envelope was normal.

#### 3.1.2 Patient 2

Histological examination revealed compact orthohyperkeratosis, vacuolated granular cells with sparse keratohyalin granules, acanthosis, papillomatosism and perivascular infiltrates (Figure 2d). No obvious neutrophilic infiltration was observed in the epidermis. The EM showed stratum corneum thickening, filaments tearing (Figure 2e) (red arrowhead), the cytoplasm and nucleus edema of hyaline layer cells, superficial granular cells containing characteristic intranuclear granules (Figure 2f) (red arrowhead). The cornified cell envelope was normal.

### 3.2 Mutation screening for GJA1, GJB3, and GJB4 genomic sequences

**Figure 3:**
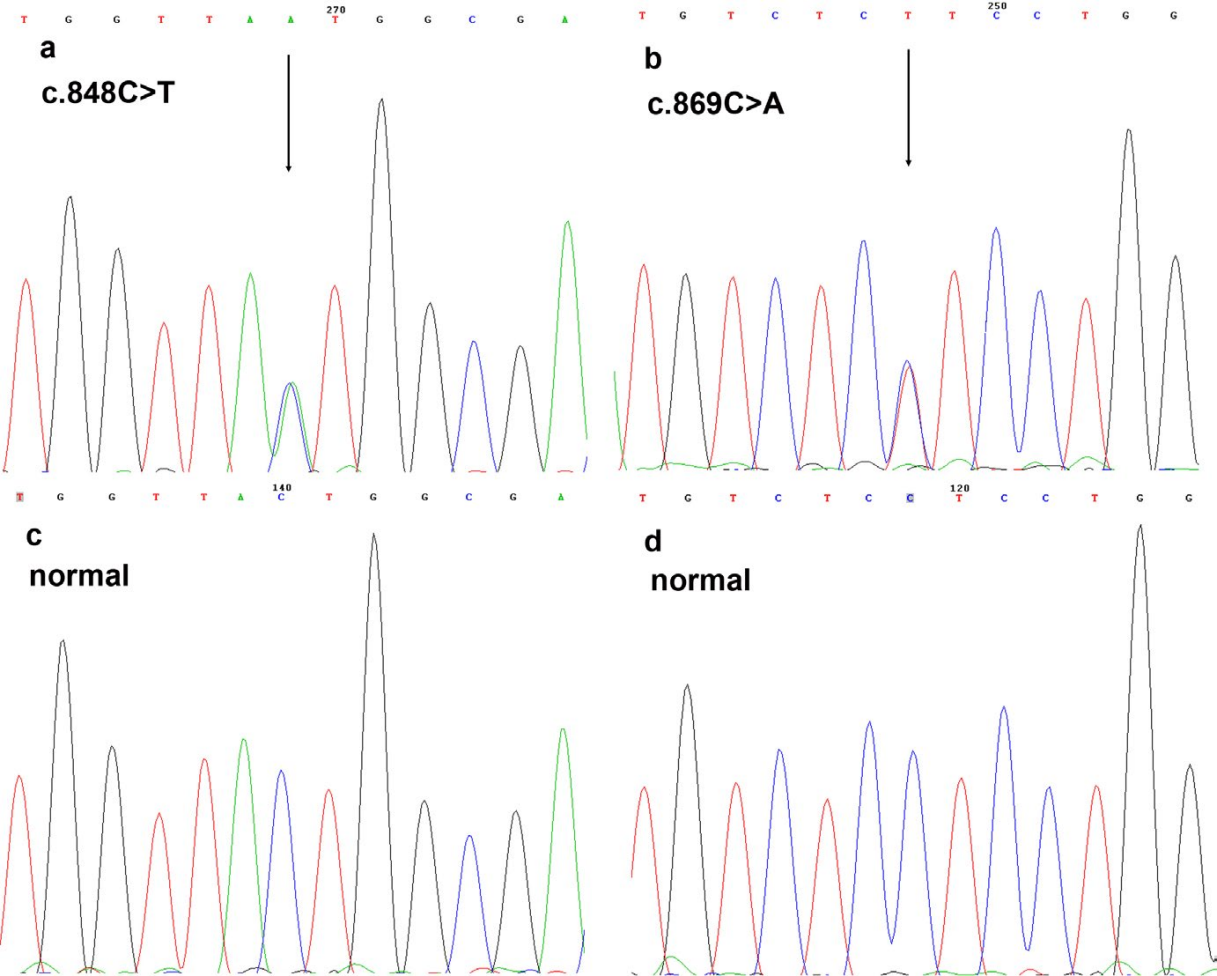
Sequence analysis of GJA1 cDNA of the patient with EKVP and an unaffected control. (a) Sequencing results demonstrating a de novo missense mutation c.848C > T (arrows), (b) and a de novo missense mutation c.869C > A (arrows) in exon 2. (c, d) Part of the wild‐type sequence in exon 2 of GJA1

Direct sequencing of all coding and exon–intron boundary sequences identified a novel heterozygous missense mutation in exon 2 of the GJA1 gene (NM_000165.4), designated as c.848C > T (Figure 3a), in both patients. This mutation, c.848C > T resulted in a change from a proline (CCT) residue at 283 to a changed leucine (CTT) residue (p.P283L). This study was also identified a novel heterozygous missense mutation in exon 2 of the GJA1 gene, designated as c.869C > A (Figure 3b), in the patient 1 (Figure 2a). The mutation, c.869C > A resulted in a change from a threonine (ACT) residue at 290 to a changed asparagine (AAT) residue (p.T290N).

These mutations have not been reported in literatures or recorded in mutation databases. Unaffected individuals in their families and 100 unrelated controls did not show these changes (Figure 3c,d). No mutation was detected in the GJB3 and GJB4 genes in the patients.

### 3.3 Immunohistochemistry and immunofluorescence

**Figure 4:**
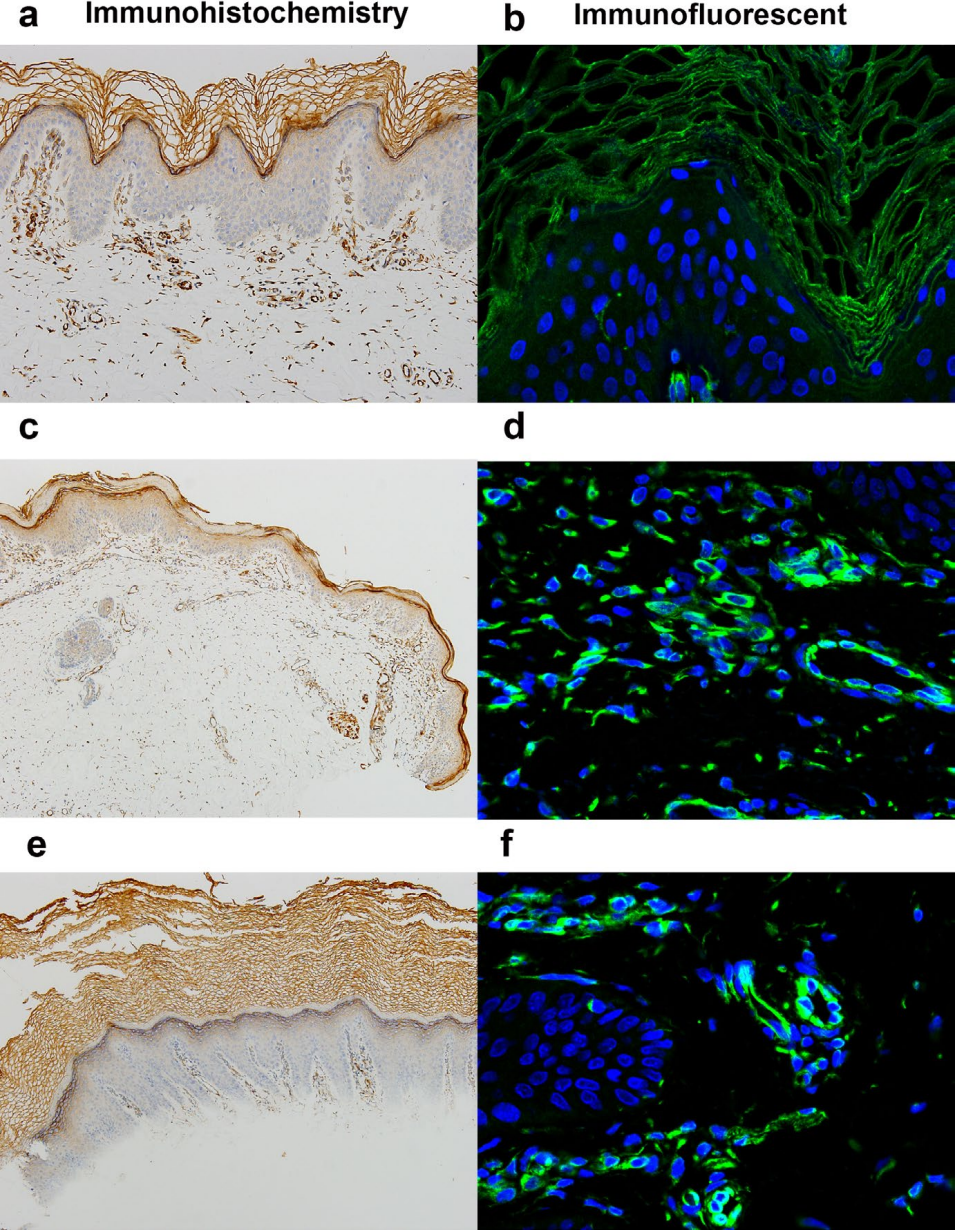
Immunohistochemistry and immunofluorescent staining of Cx43. (a, b) In control healthy skin, immunosignals of anti‐Cx43 were expressed in the intracellular junctions of keratinocytes in the stratum corneum and granular layer (×100, DAB stain, ×200). (c) In lesion of patient 1, immunosignals of anti‐Cx43 were strongly expressed in the stratum corneum, granular layer and spinous layer, whereas basal layer show low expression of Cx43 (×100, DAB stain). (d) Cx43 protein was mainly expressed in the cytomembrane and cytoplasm (×200). (e) In lesion of patient 2, immunosignals of anti‐Cx43 were strongly expressed in the stratum corneum and granular layer (×100, DAB stain). (f) Cx43 protein was mainly expressed in the cytomembrane and cytoplasm (×200)

In control healthy skin, immunosignals of anti‐Cx43 were expressed in the intracellular junctions of keratinocytes in the stratum corneum and granular layer (Figure 4a,b). In lesion of the boy, immunosignals of anti‐Cx43 were strongly expressed in the stratum corneum, granular layer and spinous layer, whereas basal layer show low expression of Cx43 (Figure 4c). Cx43 protein was mainly expressed in the cytomembrane and cytoplasm (Figure 4d). In lesion of the girl, immunosignals of anti‐Cx43 were strongly expressed in the stratum corneum and granular layer (Figure 4e). Cx43 protein was mainly expressed in the cytomembrane and cytoplasm (Figure 4f). The cytoplasmic mislocalization was more severity in the boy than that in the girl.

## 4 DISCUSSION

In this study, we report two patients with EKVP. The boy showed symmetrical erythematous keratotic erythema, plaques on the face, neck, elbows, wrists, limbs, knees, inguinal region, hands, and feet. The girl showed symmetrical erythematous, plaques on the hands, feet, wrists, and ankles. The symptoms of the boy were more severity than the girl. The general histological profile of EKVP is manifested by hyperkeratosis, slight acanthosis and papillomatosis, and some perivascular infiltration of lymphocytes and histiocytes. Vacuolation of granular cells could be observed in some cases (Zhang et al., 2010). The histological feather of our patients consistent with EKVP. At the ultrastructural level, our patients showed filaments tearing and filaments of interstellar clouds and intranuclear granules. The clinical and histologic findings were consistent with the diagnosis of EKVP. By Sanger sequencing, we identified two previously unreported de novo missense mutations in the GJA1 gene, c.848C > T p.(P283L) and c.869C > A p.(T290N) in the boy and the girl. These heterozygous mutations in GJA1 cause a consistent clinical phenotype of normal skin at birth which develops symmetrical erythematous on the hands and feet at age of 1–10 months, with progression to keratotic erythema, plaques. Our results showed that de novo GJA1 mutation could lead to a mild clinical phenotype or a severe clinical phenotype (see Table 1). The boy has compound heterozygous mutations in the GJA1 gene associated with a severe clinical phenotype, whereas the girl has single heterozygous mutation associated with a mild clinical phenotype. Our results thus reveal a nice correlation between the compound heterozygous mutations and the clinical severity.

**Table 1:** Features of all EKVP individuals with GJA1 mutations

Patient ID	Origin	Gender	Age	Onset (month)	Affected sites	Mutation type	Nucleotide mutation		Protein alteration	Cx43 location	Cases	References
1	American	M	32 months	5	Dorsal hands, arms, legs, and face	Heterozygous	c.681A > T	p.E227D		—	Sporadic	Lynn M. Boyden
2	Guatemala	F	6 years	6	Dorsal hands, palms and soles, axillae, elbows, and inner thighs, cheeks, and upper chest	Heterozygous	c.681A > T	p.E227D		Cytoplasmic localization	Sporadic	Lynn M. Boyden
3	American	F	30 years	6	Knees, elbows, hands, feet, legs, and arms	Heterozygous	c.131C > T	p.A44V		Cytoplasmic localization	Sporadic	Lynn M. Boyden
4	China	M	12 years	1	Face, neck, elbows, wrists, limbs, knees, inguinal region, hands, and feet	Compound heterozygous	c.848C > T,c.869C > A	p.P283L,p.T290N		Cytomembrane and cytoplasmic localization	Sporadic	This study
5	China	F	7 years	10	Hands, feet, wrists, and ankles	heterozygous	c.869C > A	p.T290N		Cytomembrane and cytoplasmic localization	Sporadic	This study

GJA1 located in chromosome 6q22. GJA1 is the most ubiquitously expressed connexin isoform in mammalian tissues. It forms intercellular gap junction (GJ) channels, enabling adjacent cells to communicate both electrically and metabolically (Sun et al., 2018). The GJA1 gene has two exons separated by an 11‐kb intron, and the complete coding region is included in the last exon (Paznekas et al., 2009; Willecke et al., 2002). GJA1 encodes the transmembrane protein Cx43 (Skerrett & Williams, 2017). The Cx43 protein consists of four transmembrane domains, two extracellular loops, one intracellular loop, and the intracellular amino and carboxy termini, and the carboxy termini domain appears to be the primary region that becomes phosphorylated (Alexander & Goldberg, 2003; Solan & Lampe, 2009). Six connexins generate a connexon. A connexon of one cell lines up with a connexon of an opposing cell, building a gap junction that mediates the intercellular transport of ions and molecules up to 1 kDa without exposure to the extracellular environment (Esseltine & Laird, 2016; Laird, 2014). Mutations in GJA1 can cause oculodentodigital dysplasia (ODDD), characterized by soft tissue fusion of the digits, abnormal craniofacial bone development, small eyes, and loss of tooth enamel (Laird, 2006; Pizzuti et al., 2004). Previous study showed that the carboxyl terminus, the largest domain of Cx43, has findings of only two mutations associated with ODDD, the lowest proportion of any domain (Cella et al., 2006; Paznekas et al., 2003; Van Steensel et al., 2005). Subjects are without features of ODDD caused by over 70 other GJA1 mutations, and the A44V (Boyden et al., 2015), E227D (Boyden et al., 2015), P283L, and T290N mutations were observed in five unrelated EKVP subjects (see Table 1), suggesting that EKVP pathology may be restricted to a small number of GJA1 mutation sites. The A44V (Boyden et al., 2015), E227D (Boyden et al., 2015), P283L and T290N mutations mapped to the extracellular loops, transmembrane domains, carboxy termini and carboxy termini of Cx43, respectively. The C‐terminal domain is involved in binding numerous members of the gap junction proteome, as well as being the substrate for several protein kinases (Laird, 2010; Solan & Lampe, 2009). Cx43 is the most widely expressed connexin being present in at least 34 tissues and 46 cell types (Laird, 2014; Solan & Lampe, 2009). Cx43 is the predominant connexin in human epidermis and in cultures of human keratinocytes 21 (Fitzgerald et al., 1994). In normal human skin, Cx43 is mainly expressed in the intracellular junctions of the upper more differentiated layers, whereas basal keratinocytes show low expression of total Cx43 (Richards et al., 2004). Our immunostaining shows that GJA1 mutations P283L and T290N lead to Cx43 mislocalization. In control healthy skin, immunosignals of anti‐Cx43 were expressed in the cytomembrane of keratinocytes in the stratum corneum and granular layer. In lesion of patients, immunosignals of anti‐Cx43 do not localize to the intracellular junctions, demonstrating cytoplasmic localization. The cytoplasmic mislocalization shows a more severe phenotype in the boy than that in the girl.

In conclusion, we have identified two de novo mutations in GJA1 gene in two unrelated individuals. Our finding expands the mutant spectrum of GJA1 gene and adds new understanding of the genotype‐phenotype correlation. Identification of more individuals carrying new variants in GJA1 gene is needed to fully characterize the full phenotypic spectrum associated with EKVP.

## CONFLICT OF INTEREST

The authors state no conflict of interests.
